# Regional perinatal mortality differences in the Netherlands; care is the question

**DOI:** 10.1186/1471-2458-9-102

**Published:** 2009-04-14

**Authors:** Miranda Tromp, Martine Eskes, Johannes B Reitsma, Jan Jaap HM Erwich, Hens AA Brouwers, Greta C Rijninks-van Driel, Gouke J Bonsel, Anita CJ Ravelli

**Affiliations:** 1 Department of Medical Informatics, Academic Medical Center, University of Amsterdam, Amsterdam, the Netherlands; 2 Department of Clinical Epidemiology, Biostatistics and Bioinformatics, Academic Medical Center, University of Amsterdam, Amsterdam, the Netherlands; 3 Department of Obstetrics and Gynaecology, University Medical Center Groningen, Groningen, the Netherlands; 4 Department of Neonatology, University Medical Center Utrecht, Utrecht, the Netherlands; 5 Department of Obstetrics and Gynaecology, Academic Medical Centre, Amsterdam, the Netherlands; 6 Department of Health Policy and Management, Erasmus Medical Center, Rotterdam, the Netherlands

## Abstract

**Background:**

Perinatal mortality is an important indicator of health. European comparisons of perinatal mortality show an unfavourable position for the Netherlands. Our objective was to study regional variation in perinatal mortality within the Netherlands and to identify possible explanatory factors for the found differences.

**Methods:**

Our study population comprised of all singleton births (904,003) derived from the Netherlands Perinatal Registry for the period 2000–2004. Perinatal mortality including stillbirth from 22^+0 ^weeks gestation and early neonatal death (0–6 days) was our main outcome measure. Differences in perinatal mortality were calculated between 4 distinct geographical regions North-East-South-West. We tried to explain regional differences by adjustment for the demographic factors maternal age, parity and ethnicity and by socio-economic status and urbanisation degree using logistic modelling. In addition, regional differences in mode of delivery and risk selection were analysed as health care factors. Finally, perinatal mortality was analysed among five distinct clinical risk groups based on the mediating risk factors gestational age and congenital anomalies.

**Results:**

Overall perinatal mortality was 10.1 per 1,000 total births over the period 2000–2004. Perinatal mortality was elevated in the northern region (11.2 per 1,000 total births). Perinatal mortality in the eastern, western and southern region was 10.2, 10.1 and 9.6 per 1,000 total births respectively. Adjustment for demographic factors increased the perinatal mortality risk in the northern region (odds ratio 1.20, 95% CI 1.12–1.28, compared to reference western region), subsequent adjustment for socio-economic status and urbanisation explained a small part of the elevated risk (odds ratio 1.11, 95% CI 1.03–1.20). Risk group analysis showed that regional differences were absent among very preterm births (22^+0 ^– 25^+6 ^weeks gestation) and most prominent among births from 32^+0 ^gestation weeks onwards and among children with severe congenital anomalies. Among term births (≥ 37^+0 ^weeks) regional mortality differences were largest for births in women transferred from low to high risk during delivery.

**Conclusion:**

Regional differences in perinatal mortality exist in the Netherlands. These differences could not be explained by demographic or socio-economic factors, however clinical risk group analysis showed indications for a role of health care factors.

## Background

Perinatal mortality is an important indicator of health and the quality of health care [1]. Countries or regions are often compared using perinatal mortality rate. The position of the Netherlands in international comparative research is unfavourable. In 2003 the results of the PERISTAT study showed that Dutch perinatal mortality for the year 1999 was substantially higher compared to other European countries (stillbirth rate of 7.4 per 1,000 total births and early neonatal mortality of 3.5 per 1,000 live births) [1,2].

The observed differences in perinatal mortality across Europe are difficult to explain unequivocally because of the many potential explanations like variation in registration practices, differences in definitions, and variation in demographic structure [3]. On the national level, fair comparisons can be achieved more easily. Dutch public health policies aim to reduce national health inequalities if existent [4]. It is unknown whether the current Dutch perinatal mortality is uniformly distributed across the country; differences have been reported based on civil data in the early eighties [5,6]. Although the Netherlands is a small country, regional variation exists in the degree of urbanisation, the number of immigrants and to a lesser extent, in socio-economic status. Regional variation in mortality from other causes like cardio-vascular diseases and cancer has been reported before [7-9].

Factors related to regional differences in perinatal outcome reported in other European countries after adjustment for demographic factors, include population density [10], access and use of health services [11], income level and social inequality [12,13] and excess risk for certain conditions [14].

The objective of this study is to examine whether regional differences in perinatal mortality in the Netherlands exist for the period 2000–2004, and whether these differences persist after taking into account various risk factors that have been linked to regional variation in perinatal mortality.

## Methods

### Data source

Data from the Netherlands Perinatal Registry (PRN) 2000–2004 were used. The PRN is a database that contains the linked data from three registries: the national obstetric database by midwives (LVR-1 registry), the national obstetric database by gynaecologists (LVR-2 registry) and the national neonatal/paediatric database (LNR registry) [15]. The PRN registry contains comprehensive data on pregnancy, provided care (interventions, referrals) and pregnancy outcomes [16]. The coverage of the PRN is about 96% of all deliveries in the Netherlands. All variables are recorded by the caregiver during prenatal care, delivery and neonatal, lying-in period. The data are annually sent to the national registry office, where a number of range and consistency checks are conducted. Data on socio-economic status (SES) on the postal code level was obtained from The Netherlands Institute for Social Research (SCP).

### Study population

The population for this study consisted of all singleton births born between 2000 and 2004 from 22.0 gestational weeks onwards. Gestational age was based on ultrasound or last menstrual period. If gestational age was unknown, children with a birth weight below 500 grams were excluded in accordance with the World Health Organization reporting criteria [17].

### Outcome measures

Perinatal mortality was our primary outcome measure. Perinatal mortality is defined as the sum of stillbirth (≥ 22.0 gestational weeks) and early neonatal mortality (deaths of live born children during the first week of life). Stillbirth rate and perinatal mortality rate were both calculated per 1,000 total births. Early neonatal mortality was calculated per 1,000 live births. Apart from mortality, the following mediating outcome measures were also analysed: preterm delivery (<32.0 weeks gestational age), low birth weight (<1500 gram) and low APGAR score after five minutes (APGAR score <4).

### Provinces and regions in the Netherlands

**Figure 1:**
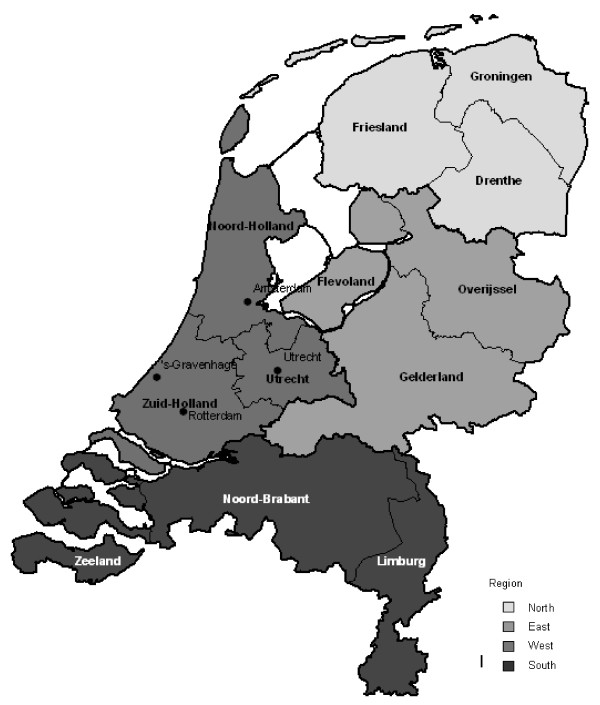
**Provinces and regions in the Netherlands**.

Regional differences in perinatal mortality were analysed on a province level and on a regional level. The Netherlands is formally divided into 12 provinces (see Figure 1), which form regional administrative units in between municipalities and the national government. The provinces were grouped into 4 regions based on their geographical position: Northern region (Groningen, Friesland, Drenthe), eastern region (Overijssel, Gelderland, Flevoland), western region (Utrecht, Noord-Holland, Zuid-Holland) and southern region (Zeeland, Noord-Brabant, Limburg). The northern region is the most rural area in the Netherlands, while the western region is most urbanised (the 4 largest cities are displayed in figure 1). The province of each woman was based on her registered postal code (4 digits) in the registry. Women with an unknown or invalid postal code (0.2%) were removed from the analyses.

### Population characteristics

Demographic characteristics of included women were compared across regions including maternal age, parity and ethnicity. Maternal age was categorised into <20 years, 20–34 years and ≥35 years. Parity was categorised into 0 (first birth), 1 (second birth) and 2+ (third or higher birth). Ethnicity is ascribed by the woman's care provider. For this study, we differentiated between Western (native Dutch and other Westerners) and non-Western (including different ethnic groups like African/Surinamese Creole, Surinamese Hindustani, Moroccan and Turkish).

In addition, data on socio-economic status (SES) were obtained from The Netherlands Institute for Social Research/SCP on postal code level. Using the woman's postal code (4 digits) these data could be linked to the perinatal registry file. The SES score is based on mean income level, the percentage of households with a low income, the percentage of inhabitants without a paid job and the percentage of households with on average a low education in a postal code area [18]. The continuous SES score was for our purpose categorised into a high, middle and low group based on percentile ranges (≤ 25^th ^percentile, middle, > 75^th ^percentile). The data on socio-economic status were available for the year 2002. The categorised score was applied to the total population for the period 2000–2004 as large changes in SES score for a postal code area within two years are unlikely. By using the same postal code we could add the degree of urbanisation (number of addresses per square kilometre), a number which is routinely available. The degree of urbanisation was categorised into three groups: very rural, rural to urban and very urbanised.

### Clinical characteristics

Besides population characteristics, we analysed regional variation in health care services. We geographically compared the mode of delivery and risk selection at start of delivery. Risk selection is an important feature of the Dutch obstetric system [19]. Healthy women with an uncomplicated obstetric history and/or pregnancy remain under the care of the primary level midwife and are selected as at low risk at start of delivery. In that case a woman can choose to deliver at home or at the hospital, both under supervision of the midwife. If complications occur the woman is selected as high risk and is referred to an obstetrician at the secondary or tertiary level. We analysed the risk selection status at the start of delivery.

### Statistical analysis

Differences in adverse outcomes by region and province were tested by Chi-Square test using all other regions/provinces as the reference category. Differences in population characteristics by region were tested by Chi-Square test. After describing crude mortality, logistic regression modelling was used to estimate differences in perinatal mortality between regions after adjustment for socio-demographic factors. All previously described factors were added to the model in two successive steps. First we adjusted only for demographic factors parity, maternal age and ethnicity, parity and maternal age were included as categorical variables with the category with the lowest mortality risk as reference. In the second model we additionally adjusted for the degree of urbanisation and SES. In both models we included the year of registration to incorporate changes in perinatal mortality over time. The strength of the association between potential predictors and perinatal mortality are expressed as odds ratios (OR) with 95% confidence intervals (CI).

For further interpretation of possible regional differences in perinatal mortality, the perinatal mortality risk for five clinical relevant risk groups was examined by region. These groups represent distinct clinical entities with different patterns of care based on mediating risk factors gestational age and severe congenital anomalies. Severe congenital anomalies were defined as anomalies which are either highly fatal or as anomalies potentially detectable by ultrasound and severe enough for optional late termination of pregnancy. The five groups are very preterm births (22^+0^–25^+6 ^weeks), severe congenital anomalies, preterm births (26^+0^–31^+6 ^weeks) without severe congenital anomalies, preterm births (32^+0^–36^+6 ^weeks) without severe congenital anomalies and term births (≥ 37^+0 ^weeks) without severe congenital anomalies.

All analyses were performed using SAS for Windows (version 9.1, SAS Institute Inc., Cary, NC, USA).

## Results

During the period 2000–2004, there were 904,003 singletons births in the Netherlands. Nearly half of all births were in the urbanised western region (46.1%) and only 9.7% in the northern more rural region (see table 1). The overall perinatal mortality in the Netherlands in the period 2000–2004 was 10.1 per 1,000 total births. The northern region has the highest perinatal mortality rate with 11.5 and 11.9 per 1,000 total births in provinces Groningen and Friesland respectively. The southern region had the lowest perinatal mortality rate, with lowest rates in provinces Noord-Brabant and Limburg: 9.5 and 9.4 per 1,000 total births. The perinatal mortality rate in the northern region was significantly higher than in the other regions (Chi-square p-value < 0.01). Both stillbirth and early neonatal mortality were high in the northern provinces. The proportion of preterm births and children with a low birth weight was also significantly higher in the northern region (1.2% and 1.2% respectively).

**Table 1:** Adverse outcomes by region and province for singletons in the period 2000–2004.

**Region/Province**	**Total ** **number** **of children**		**Perinatal ** **mortality** **≥22**^+0^**wks – 6 d**	**Still birth ** **≥22**^+0^**wks**	**Early ** **neonatal ** **mortality** **(0–6 d)**	**Preterm** **birth** ** <32**^+0^**wks**	**Low** **birth** **weight** ** <1500 gram**	**Low** **APGAR** ** score <4**
	**#**	**%**	**‰**	**‰**	**‰**	**%**	**%**	**%**
**Region**								
North (N)	87,857	9.7	11.2*	7.9	3.3	1.2*	1.2*	0.3
East (E)	200,158	22.1	10.2	7.3	2.9	1.1	1.1	0.3
West (W)	416,768	46.1	10.1	7.2	2.9	1.1	1.1	0.3
South (S)	199,220	22.0	9.6*	6.9	2.7	1.1	1.1	0.3
**Province**								
Groningen-N	30,200	3.3	11.5	8.4	3.1	1.4*	1.4*	0.4
Friesland-N	31,554	3.5	11.9*	8.1	3.9*	1.3*	1.3	0.3
Drenthe-N	26,103	2.9	9.9	7.1	2.8	1.1	1.0	0.2
Overijssel-E	65,980	7.3	10.2	7.3	2.8	1.1	1.1	0.3
Gelderland-E	113,496	12.6	10.1	7.2	2.9	1.1	1.1	0.3
Flevoland-E	20,682	2.3	10.9	7.9	3.1	1.2	1.3	0.3
Utrecht-W	73,645	8.1	10.2	6.7	3.5*	1.0	1.0*	0.3
Noord-Holland-W	149,009	16.5	9.8	7.1	2.8	1.2	1.1	0.3
Zuid-Holland-W	194,114	21.5	10.3	7.5	2.8	1.1	1.1	0.3
Zeeland-S	15,363	1.7	10.9	7.5	3.4	0.9	1.1	0.3
Noord-Brabant-S	130,973	14.5	9.5	6.8	2.7	1.1	1.1	0.3
Limburg-S	52,884	5.8	9.4	6.8	2.6	1.1	1.2	0.3

**Total**	**904,003**	**100**	**10.1**	**7.2**	**2.9**	**1.1**	**1.1**	**0.3**

* Significantly different (p < 0.01) from all other regions/provinces excluding the region/province itself.

The northern region had the largest proportion of women from rural areas (41.8%) and with a low SES score (38.0%) and the lowest proportion of non-western women (7.2%) (Table 2). The western region had the highest proportion of women aged above 35 years (20.6%), with non-western ethnicity (22.7%), with a high SES score (32.9%) and living in urban areas (36.4%). All these regional differences were statistically significant.

**Table 2:** Population characteristics by region for the period 2000–2004.

	**North**		**East**		**West**		**South**		**Chi-Square**
	**#**	**%**	**#**	**%**	**#**	**%**	**#**	**%**	
Number of singleton pregnancies	87,857	100	200,158	100	416,768	100	199,220	100	
Maternal age									
<20 years	1,805	2.1	3,258	1.6	8,179	2.0	3,344	1.7	p < 0.0001
20–34 years	71,651	81.6	161,834	80.9	322,647	77.4	162,188	81.4	
>= 35 years	14,401	16.4	35,066	17.5	85,942	20.6	33,688	16.9	
Parity									
Nulliparous	40,459	46.1	89,976	45.0	197,514	47.4	93,654	47.0	p < 0.0001
Parity 1	32,160	36.6	72,213	36.1	143,731	34.5	72,746	36.5	
Parity 2+	15,238	17.3	37,969	19.0	75,523	18.1	32,820	16.5	
Ethnicity non-western	6,356	7.2	20,810	10.4	94,415	22.7	23,206	11.6	p < 0.0001
Heavy smoking	727	0.8	1,102	0.6	1,678	0.4	1,036	0.5	p < 0.0001
Urbanisation									
Very urban	6,332	7.2	6,107	3.1	152,444	36.6	10,505	5.3	p < 0.0001
Middle	44,806	51.0	149,537	74.7	232,546	55.8	138,893	69.7	
Very rural	36,719	41.8	44,514	22.2	31,778	7.6	49,822	25.0	
SES									
High	13,175	15.0	46,103	23.0	137,766	33.1	40,413	20.3	p < 0.0001
Middle	41,321	47.0	112,929	56.4	163,727	39.3	120,345	60.4	
Low	33,361	38.0	41,126	20.5	115,275	27.7	38,462	19.3	

Table 3 shows that women from the northern region had a significantly higher perinatal mortality risk compared to the western region (unadjusted OR 1.11, 95% CI 1.03–1.19). The western region was set as reference area because the perinatal mortality risk was the same as the overall rate. After adjustment for demographic factors (maternal age, parity and ethnicity), the women in the northern region (OR 1.20, 95% CI 1.12–1.28) and eastern region (OR 1.08, 95% CI 1.02–1.14) had a significantly higher perinatal mortality risk compared to women in the western region. Living in a very urban area and having a low SES score were significant risk factors for perinatal mortality, while a high SES score lowered the risk. Subsequent adjustment for urbanisation degree and social-economic status explained a small part of the excess risk in the northern region (OR 1.11, 95% CI 1.03–1.20). Living in an very urban area was no longer a risk factor in adjusted model II.

**Table 3:** Perinatal mortality (22^+0 ^weeks – 6 days) risk per region after adjustment for risk factors.

	**Unadjusted**		**Adjusted Model I**^#^		**Adjusted Model II**^†^	
	**OR**	**95% CI**	**OR**	**95% CI**	**OR**	**95% CI**
Region						
North	1.11	1.03–1.19	1.20	1.12–1.28	1.11	1.03–1.20
East	1.01	0.96–1.07	1.08	1.02–1.14	1.04	0.98–1.10
West*	1.00	reference	1.00	reference	1.00	reference
South	0.95	0.90–1.00	1.01	0.95–1.06	0.97	0.91–1.03
Maternal age						
< 20 years	1.68	1.49–1.91	1.38	1.22–1.57	1.36	1.20–1.54
20–34 years	1.00	reference	1.00	reference	1.00	reference
≥ 35 years	1.23	1.17–1.30	1.28	1.21–1.35	1.29	1.23–1.36
Parity						
Parity 0	1.40	1.33–1.47	1.42	1.35–1.49	1.42	1.35–1.49
Parity 1	1.00	reference	1.00	reference	1.00	reference
Parity 2+	1.44	1.36–1.53	1.32	1.24–1.41	1.31	1.23–1.40
Ethnicity						
Western	1.00	reference	1.00	reference	1.00	reference
Non-Western	1.42	1.35–1.50	1.43	1.36–1.50	1.37	1.29–1.45
Urbanization						
Very urban	1.10	1.05–1.16			0.92	0.86–0.98
Middle	1.00	reference			1.00	reference
Very rural	1.01	0.96–1.07			1.03	0.98–1.10
SES						
Low	1.23	1.18–1.30			1.15	1.09–1.21
Middle	1.00	reference			1.00	reference
High	0.90	0.85–0.95			0.91	0.86–0.96

* Region west was set as reference area as the perinatal mortality rate of this region was equal to the overall rate.

^# ^Model I was adjusted for maternal age, parity and ethnicity.

^† ^Model II was an extension of model I with additional adjustment for degree of urbanization and SES.

The health services patterns also exhibited regional differences (Table 4). The northern region had the lowest number of spontaneous deliveries (72.9% versus 75.6% in the western region) and the lowest number of women selected as low risk at start of delivery (42.7% versus 50.2% in the western region). The percentage of home births was 19.7% in the northern region versus 23.0% in the western region and in the eastern region the percentage of home births was as high as 30.4%. There were only small variations in the percentage of women transferred from low risk to high risk during delivery (11.2% in the northern region and 12.7% in the western region). In the northern region most deliveries take place under supervision of an obstetrician (57%). The northern region has the lowest number of hospitals and only 1 tertiary hospital.

**Table 4:** Description of health care factors by region.

	**North**		**East**		**West**		**South**	
	**#**	**%**	**#**	**%**	**#**	**%**	**#**	**%**
**Mode of delivery**								
Spontaneous	64,010	72.9	151,816	75.8	315,282	75.6	149,366	75.0
Elective Caesarean Section	6,046	6.9	12,010	6.0	24,416	5.9	12,826	6.4
Instrumental vaginal delivery	10,680	12.2	22,019	11.0	45,538	10.9	21,018	10.6
Emergency Caesarean Section	7,121	8.1	14,313	7.2	31,532	7.6	16,010	8.0
								
**Care at start of delivery**								
low risk selection	37,506	42.7	103,069	51.5	209,376	50.2	92,418	46.4
low risk & home delivery	17,337	19.7	60,869	30.4	95,724	23.0	46,918	23.6
low risk & hospital delivery	10,349	11.8	18,157	9.1	60,656	14.6	20,250	10.2
from low to high risk during delivery	9,820	11.2	24,043	12.0	52,996	12.7	25,250	12.7
high risk selection	50,351	57.3	97,089	48.5	207,392	49.8	106,802	53.6
								
**Number of hospitals**	15		18		39		25	
**Number of tertiary centres**	1		2		5		2	

**Total**	**87,857**	**100**	**200,158**	**100**	**416,768**	**100**	**199,220**	**100**

Among very preterm births (responsible for 28% of all perinatal deaths), the perinatal mortality risk was about the same in all regions (Table 5). The perinatal mortality risk for children with severe congenital anomalies (responsible for 12% of perinatal deaths) was higher in the northern region (204 per 1,000 births) compared to the western region (147 per 1,000). The mortality risk among preterm births 26^+0^–31^+6 ^weeks (responsible for 14% of perinatal deaths) in the northern region was lower than in the western region (233 versus 237 per 1,000). The mortality risk among preterm births 32^+0^–36^+6 ^weeks (responsible for 18% of perinatal deaths) was higher in the northern region and lower in the southern region than in the western region. For the term births (responsible for 28% of all perinatal deaths) the mortality risk was about 11% higher in the northern region compared to the western region (3.4 per 1,000 versus 3.1 per 1,000 in region west). Within the term group (≥37^+0 ^weeks), the regional mortality difference was the largest for the group of births from women transferred from low risk to high risk during delivery (4.0 per 1,000 in north versus 2.6 per 1,000 in west). The perinatal mortality risk for term births from women selected as high risk at start of delivery was similar; in both northern and western regions 5.0 per 1,000.

**Table 5:** Prevalence and mortality risk for clinical risk groups by region.

	**North**		**East**		**West**		**South**	
	**Prev**	**Mortality risk**	**Prev**	**Mortality risk**	**Prev**	**Mortality risk**	**Prev**	**Mortality risk**
**Clinical risk groups**	**%**	**‰**	**%**	**‰**	**%**	**‰**	**%**	**‰**
Very preterm births <26^+0 ^weeks	0.27	937	0.28	947	0.31	928	0.32	951
Severe congenital anomalies	0.80	204	0.81	163	0.81	147	1.02	105
Premature 26^+0^–31^+6 ^weeks	0.89	233	0.74	248	0.76	237	0.72	214
Premature 32^+0^–36^+6 ^weeks	5.14	33.6	4.77	30.2	4.75	29.6	5.26	26.6
Term ≥ 37^+0 ^weeks	92.89	3.4	93.40	3.2	93.38	3.1	92.69	2.8

**Total**	**100**	**11.2**	**100**	**10.2**	**100**	**10.1**	**100**	**9.6**

Prev = prevalence.

## Discussion

The perinatal mortality in the Netherlands for the period 2000–2004 shows regional variation, with an increased perinatal mortality in the rural northern region. The regional variation was present in both stillbirth and early neonatal mortality. The elevated risk in the northern region could not be explained by regional variation in demographic risk factors like maternal age, parity and ethnicity. Socio-economic status and urbanisation grade only explained a small part of the excess risk. Analyses focussed on clinical relevant subgroups showed regional differences were most prominent among births from 32^+0 ^weeks gestation onwards and especially among term births from women transferred from low to high risk during delivery.

Data from a period of five years could be analyzed including 904,003 pregnancies in the Netherlands. The Netherlands Perinatal Registry contains the combined information on pregnancy, childbirth and the neonatal period derived from three separate registries that have recently been linked using probabilistic record linkage techniques. This enabled us to adjust for a combination of demographic, care related and socio-economic factors in relation to perinatal outcome [16,20].

Data from general practitioners providing obstetric care were not available from the Netherlands perinatal registry. General practitioners more often provide obstetric care in rural areas, which are found in the northern region but also in the eastern and southern regions [21]. Overall the proportion of deliveries that took place under supervision of a general practitioner is estimated at 4%. However over 99% of hospital deliveries were included and a woman is transferred to an obstetrician by the general practitioner in case of high risk. This is in accordance with the finding when the perinatal registry data were linked to civil registry data in a pilot study, more foetal deaths were registered in the perinatal registry, especially the very premature foetal deaths. Medical registries suffer from entry errors by professionals as any database. Limited entry options and data checks by professionals combined with validated linkage procedures [15,22] have confined errors to a minimum. The current perinatal registry does not contain information on smoking (only reporting on heavy smoking with clear underestimation), food intake, folic acid intake, maternal education and body mass index (BMI), factors which may (partly) explain the regional differences in perinatal mortality [23-25]. Additional adjustment for BMI and smoking on a province level for women in the reproductive age (data from Statistics Netherlands and STIVORO) did not change the elevated perinatal mortality risk in the northern region (data not shown). Risk factor behaviour in pregnancy is related to both socio-economic class and ethnicity [26,27]. After adjustment for these factors the risk status of the northern region remained high; therefore we believe that regional variation in unmeasured risk factors is unlikely to explain the observed differences in mortality in our adjusted models. One could challenge the use of the SES score on a neighbourhood level rather than on the individual level. However, previous research on socio-economic inequalities have demonstrated that this is a valid approach [28,29].

This is the first time that regional differences in perinatal mortality were studied in the Netherlands using the national linked perinatal registry data on 904,003 pregnancies. Previous regional analyses were based on aggregate data on 11 provinces for the period 1979–1982 [6] and on 40 economic sub-regions for the period 1980–1984 [5], rather than on individual data. Treffers et al. found differences in the percentage of hospital deliveries (versus home deliveries) per province, but could not relate this to regional perinatal mortality rate [6]. We also found differences in hospital deliveries per region and found that the regional differences were most pronounced among term women transferred from low to high risk during delivery. Mackenbach et al. reported perinatal mortality rate to depend on mean income, part of the population living in a large municipality and the presence of a level-two hospital [5]. We applied individual demographic adjustment, and used more refined variables to account for SES and urbanisation. We had access to urbanisation and SES on the neighbourhood level based on postal code, which showed large variation between regions. Social factors have been reported as explanatory factors for perinatal mortality differences [12,13], however adjustment for SES and urbanisation only explained a small part of the excess risk in the northern region in our study. The presence of fewer hospitals in the northern region may have played a role. The differences in regional perinatal mortality are sizable, and consistent with recently observed mortality differences for other causes (cardiovascular, cancer) [30].

Regional variation in health outcomes can be caused by variation in incidence of complications and/or variation in prognosis. Health status of the women and preventive and obstetric care if applicable can influence incidence and prognosis. Both stillbirth and neonatal mortality were increased in the northern region, which indicates a role for factors common to both. Population composition factors and environmental risk factors, undetected by the direct and indirect adjustment factors can be present, but their presence is less likely given the adjustments. The analyses suggest that prevention and care factors may have played a role. Potential candidates are the following.

The uptake of prevention (general – smoking, specific – folic acid, screening) may be less or the intensity or effectiveness of health services may be lower [31]. As differential access will have been partly covered by the adjustment factor urbanisation, explanations at the care level are more likely. The number of clinical facilities in the northern region is smaller, and only 1 tertiary centre is available. Perhaps intensity and quality of preventive and delivery care is less in areas with low population density [32,33]. The elevated mortality risk for children with congenital anomalies in the northern region (while prevalence was similar) might also point to differences in care shortly after birth. Late neonatal mortality (7–27 days) showed the same regional pattern, excluding mortality differences by different care management during the first week and subsequent delay of mortality. As an increased mortality risk in the northern region is present in both preterm and term births also differences in hospital supply services (obstetrical, neonatal) has to be considered, and delay due to the on average larger travelling distances in case of intended home births with an emerging risk requiring hospital admission. The group transferred from low to high risk during delivery is at higher risk for perinatal mortality than women who deliver under care of a midwife completely [16,34]. For term births the regional mortality differences were most pronounced in the group transferred from low to high risk during delivery, possibly indicating a role for travel distance. Further exploring the role of care factors rests on more detailed analysis of clinical risk groups for perinatal mortality and of stillbirth and neonatal mortality separately, but also on audit studies [35,36]. Audit studies could also provide information on causes of death, which is not registered in the current PRN registry. Against the background mortality observed in the other regions, the observed mortality in the northern region of 11.2 per 1,000 births and about 17,500 deliveries annually, this excess risk in the northern region accounts for about 19 deaths a year.

## Conclusion

In conclusion, our study revealed persistent adverse perinatal outcome in the northern part of the Netherlands even after adjustment for demographic and socio-economic factors. Analysis of clinical risk groups showed perinatal mortality differences were most pronounced among children with severe congenital anomalies and among term births from women transferred from low to high risk during delivery. The results provide an incentive to explore the role of health care factors, both at the prenatal and delivery stage of care.

## Competing interests

The authors declare that they have no competing interests.

## Authors' contributions

AR, MT, ME, JR and GB contributed to the conception and design of the study. JJE, HB and GR were involved in acquisition of the data. MT and AR conducted the analyses and MT drafted the manuscript. AR, GB, JR and ME provided advice on the analysis and interpretation of the data. All authors critically revised the draft versions and approved the final version of the manuscript.

## Pre-publication history

The pre-publication history for this paper can be accessed here:


